# Analysis of Hepatobiliary Disorder Reports Associated With the Use of Herbal Medicines in the Global Suspected ADR Database Vigibase

**DOI:** 10.3389/fphar.2019.01326

**Published:** 2019-11-06

**Authors:** Florence van Hunsel, Sonja van de Koppel, Souad Skalli, Andrea Kuemmerle, Lida Teng, Jia-bo Wang, Joanne Barnes

**Affiliations:** ^1^The Netherlands Pharmacovigilance Centre Lareb, ‘s-Hertogenbosch, Netherlands; ^2^Microbiology and Molecular Biology Team, Center of Plant and Microbial Biotechnology, Biodiversity and Environment, Faculty of Sciences, Mohammed V University in Rabat, Rabat, Morocco; ^3^Department of Medicine, Swiss Tropical and Public Health Institute, Basel, Switzerland; ^4^University of Basel, Basel, Switzerland; ^5^Department of Health Economics and Outcomes Research, Graduate School of Pharmaceutical Sciences, The University of Tokyo, Tokyo, Japan; ^6^Institute of Chinese Herbal Medicine, Beijing Integrative Medical Center for Liver Diseases, Beijing 302 Hospital of China, Beijing, China; ^7^School of Pharmacy, The University of Auckland, Auckland, New Zealand

**Keywords:** herbal medicines, hepatobiliary disorders, adverse drug reactions, VigiBase, pharmacovigilance

## Abstract

**Introduction:** Use of herbal medicines (HMs) is widespread across the world, with many people relying on HMs for their primary healthcare or using HMs in the context of a healthy life style. HMs originate from plant material and, as such, are often seen as “natural” and believed to be (relatively) safe by patients. Hepatobiliary disorders have been associated with numerous HMs.

**Aim:** This paper aims to analyze reporting patterns for hepatobiliary disorders associated with HMs use from reports submitted to the WHO global database of individual case safety reports (ICSRs) VigiBase.

**Methods:** A data extraction in VigiBase, the WHO international database of ICSR reports, was performed by the Uppsala Monitoring Centre on 2019-01-16. The dataset contained all ICSRs where an HM was identified with the UMC-assigned ATC code “V90: unspecified herbal and traditional medicine” and where the HM was classified as being either the suspected drug or an interacting drug, and containing at least one adverse reaction in the MedDRA^®^ System Organ Class (SOC) Hepatobiliary Disorders (HBD). Descriptive analyses in Excel 2013^®^ were used to determine general characteristics of the reports in the broad data set, including total number of reports, reporting country and patient characteristics. For single suspect herbal reports, reports categorized as “serious” according to CIOMS criteria (CIOMS), 2001) were extracted.

**Results:** In total, 2,483 reports describing with at least one ADR in the SOC HBD were extracted from VigiBase. In total, 780 (31.4%) reports concern only one suspect HM. However, for 188 reports of these reports (24.1%), the single suspect herbal preparation contains more than one herbal ingredient. The 592 reports for single suspect herbal preparations described a total of 764 ADRs in the SOC HBD. Jaundice was the most reported ADR for these reports.

**Conclusion:** Almost 2,500 reports for HMs and with at least one ADR coded to the MedDRA^®^ SOC HBD were retrieved from VigiBase. Of the HBD SOC HM reports, around 25% concerned a single herbal species as the suspect “drug.” Substantial issues with coding of the suspect herbal drugs were found. In-depth causality assessment of the cases is needed to draw conclusions on the strength of the relationships.

## Introduction

Herbal medicines (HMs) include herbs, herbal materials, herbal preparations (comminuted or powdered herbal materials, or extracts, tinctures and fatty oils of herbal materials) and finished or manufactured herbal products found in pharmaceutical dosage forms (tablets, capsules) (World Health Organization, 2004). Traditional medicine collectively refers to numerous different medical systems [e.g., Traditional Chinese medicine (TCM), Ayurvedic medicine, Australian Aboriginal medicine, medical herbalism, etc] that has a long history of use. It is the sum total of the knowledge, skills, and practices based on the theories, beliefs, and experiences indigenous to different cultures, whether explicable or not, used in the maintenance of health, as well as in the prevention, diagnosis, improvement or treatment of physical and mental illnesses. Most traditional medical systems make use of medicinal plants and, sometimes, other natural substances (e.g., animal parts, minerals) as a/the main approach to medical treatment. The terms complementary/alternative/non-conventional medicine are used interchangeably with traditional medicine in some countries (World Health Organization, 2004). Use of HMs and other TMs is widespread across the world, with many people relying on HMs/TMs for their primary healthcare, particularly those in low-middle income countries (WHO TCAM strategy) (World Health Organization, 2013), or using HMs in the context of a healthy life style (Ekor, 2014). The use of HMs in Europe is also widespread (Fisher and Ward, 1994; Barnes et al., 1999; de Boer et al., 2015; Kemppainen et al., 2018). HMs originate from plant material and, as such, are often seen as “natural” and believed to be (relatively) safe by patients (Vickers et al., 2006). However, HMs contain pharmacologically active constituents, and numerous HMs, or their constituents, have been associated with the occurrence of adverse drug reactions (ADRs) (Posadzki et al., 2013; de Boer et al., 2015; Venhuis et al., 2016). The occurrence of ADRs associated with HMs, including those resulting from interactions between herbal medicines (HMs) and conventional drugs, is a global public health concern (Skalli et al., 2007; Skalli and Soulaymani, 2012). Against this background, pharmacovigilance (PV) activities to identify, evaluate, and respond to signals of safety concerns associated with HMs are essential (Barnes, 2003).

PV is defined as “the science and activities relating to the detection, assessment, understanding and prevention of adverse effects or any other drug-related problem” (World Health Organization, 2019). The World Health Organization (WHO) established its Programme for International Drug Monitoring in response to the thalidomide disaster detected in 1961. Together with the WHO Collaborating Centre for International Drug Monitoring (the Uppsala Monitoring Centre in Sweden), the WHO promotes PV activities at the country level (the World Health Organization, 2019). The UMC maintains the global ADR database of individual case safety reports (ICSRs) VigiBase. This database contains over 19 million reports of ADRs related to medicines, submitted, since 1968, by member countries of the WHO Programme for International Drug Monitoring (the Uppsala Monitoring Centre, 2019).

Spontaneous ADR reporting and active surveillance conducted by national PV centers can permit rapid detection of potentially harmful combinations of medicines and HMs (Skalli and Soulaymani, 2012). However, while PV systems for conventional medicines are well-established and incorporated in healthcare and regulatory activities, this is far less the case for HMs (Barnes, 2003).

In undertaking PV for HMs, the complex characteristics of HMs, not least that each herbal ingredient typically contains hundreds of chemical constituents, batch-to-batch chemical variation of herbal ingredients and common place use of ambiguous nomenclature pose particular challenges (Dauncey et al., 2019; Shaw et al., 2012). Further difficulties arise due to the ways in which many herbal products are formulated (for example, as multi-ingredient products containing other herbal or non-herbal substances), used by patients (for example, concurrent use of multiple HM products or together with conventional drugs), and used by herbal or traditional medicine practitioners (for example, as individualized, multi-ingredient formulae, which may change with each consultation) (Barnes, 2003). A recognized limitation of spontaneous reports is that the incidence and prevalence of ADRs related to medicines are difficult to predict because of under-reporting (Hazell and Shakir, 2006); for several reasons, this is likely to be even greater for HMs than for conventional medicines (Barnes et al., 1998; Skalli and Soulaymani, 2012; Vohra et al., 2012;Necyk et al., 2014). Despite these issues, spontaneous reporting of ADRs remains the cornerstone of PV for HMs and can be used to gain insight into the safety of HMs as used in clinical practice (Skalli and Soulaymani, 2012).

Among the risks associated with HMs, hepatobiliary disorders (HBD) have been associated with numerous HMs (Real et al., 2019). HBDs are non-neoplastic or neoplastic disorders that affect the liver, bile ducts, and/or gallbladder. Representative examples of non-neoplastic disorders include hepatitis, cirrhosis, and cholangitis (The SIDER Side Effect Resource, 2015). Recognition of HBD is increasing, and the list of HMs implicated in causing these reactions has continued to grow and is of international interest (Navarro et al., 2017; Real et al., 2019). In some countries in the Asia-Pacific region, HMs and “dietary supplements” are major causes of HBD (Wong et al., 2019). Dietary supplements are often defined as products taken by mouth that contain a “dietary ingredient.” Dietary ingredients include vitamins, minerals, amino acids, and herbs or botanicals, as well as other substances that can be used to supplement the diet. Dietary supplements come in many dose forms, including tablets, capsules, powders, energy bars, and liquids (US Food and Drug Administration, 2015). A large systematic review, aiming to evaluate the clinical characteristics and outcomes of TCM-induced liver injury and to estimate the proportion of TCM-related liver injury in all drug-induced liver injuries, showed that in China, around 25% of TCMs are suspected drugs in published HBD cases. This proportion has gradually increased between 1998 and 2016 (Wang et al., 2018b). HM-related hepatotoxicity is also a common cause of drug-induced liver injury in Western countries (Stournaras and Tziomalos, 2015). In a prospective study from Iceland that included 96 patients with drug-induced liver injury between 2010 and 2011, 16% of cases were attributable to dietary supplements (Björnsson et al., 2013). The drug induced liver injury network in the United States, which upholds the largest database of HMs-related hepatotoxicity, found that between 2004 and 2013, 15.5% of cases (130 out of 839 in total) of DILI were likely caused by herbal dietary supplements. In addition, they found a temporal trend in increase in liver injury herbal dietary supplements (Navarro et al, 2014).

HMs associated with HBD include green tea (*Camellia sinensis*) extracts, ephedra (*Ephedra sinica*, also known as ma huang), black cohosh (*Actaea racemosa*), germander (*Teucrium chamaedrys*), kava (kava kava or *Piper methysticum*), blue skullcap (*Scutellaria lateriflora*), pennyroyal (*Mentha pulegium*), and HMs containing unsaturated pyrrolizidine alkaloids (Bunchorntavakul and Reddy, 2013;Garcia-Cortes et al., 2016;Real et al., 2019). Some HMs contain illicit ingredients, such as androgenic anabolic steroids in supplements promoted for body building, which may be the cause of drug-induced liver injury (DILI) rather than the labeled herbal ingredients (Garcia-Cortes et al., 2016).

## Aim

This paper aims to analyze reporting patterns for HBD associated with HMs from reports submitted to the WHO global database of ICSRs VigiBase.

## Methods

### Data Source and Definition Dataset

A data extraction in VigiBase, the WHO international database of suspected ADR reports (Linquist, 2008), was performed by the Uppsala Monitoring Centre (UMC) on 2019-01-16. The dataset date was 2019-01-01. The extracted dataset contained all ICSRs where a HM was identified with the UMC-assigned ATC code “V90: unspecified herbal and traditional medicine” and where the HM was classified by the reporter as being either suspected or interacting (reports with only concomitant HM drugs were not extracted). This ATC code is assigned to: products containing herbal ingredients only; to mixed products containing herbal ingredients and non-herbal substances (including ingredients of animal origin, e.g., deer velvet; substances such as vitamins/minerals, and occasionally conventional drugs—where such substances are co-ingredients with herbal substances, e.g., low-dose paracetamol with herbal ingredients in traditional Chinese cough/cold remedies). Mixed products that contain both herbal ingredients and conventional medicines in one product were excluded from the analysis.

Herbals are coded at national center level in reports. The level of coding can include common names, brandnames of products or botanical names. The reported herbs are coded to a “PreferredBaseName,” which is the drug active ingredient in VigiBase, which uses botanical names if possible. This entails that the reported medication name could be a common name, e.g., St John’s wort, which are then coded as Hypericum perforatum for the drug active ingredient. As there maybe multiple common names for the same botanical name, there is in theory a level of “assumption” in this coding. For our analysis, the “PreferredBaseName” in VigiBase was used. ADRs were coded by national centers contributing to VigiBase. All ICSRs in VigiBase are automatically coded with MedDRA, the Medical Dictionary for Regulatory Activities (MedDRA) coding system, which refers to a group of MedDRA terms belonging to a System Organ Class [The International Conference on Harmonisation of Technical Requirements for Registration of Pharmaceuticals for Human Use (ICH), 2017]. For this study, a subset of all HM reports based on the MedDRA System Organ Class (SOC) HBD was extracted from VigiBase.

Some other MedDRA SOCs contain ADR “preferred terms” relevant to hepatic injury, such as the terms “hepatic enzymes increased” and “alanine aminotransferase increased” in the SOC Investigations; these were not included in this analysis.

Data were extracted on: date entered in UMC database; reporting country; seriousness; patient death; report type; patient age and gender; outcome of the ADR; onset date of the ADR(s); suspect “drug”; status as suspect or concomitant drug’s; start and stop dates for “drug”; “drug” dose -usage, -administration, -route; indication for use; ADR on MedDRA SOC, -PT and -LLT level; causality as mentioned in the report; dechallenge (action taken and outcome); rechallenge (action taken and outcome).

Suspected duplicate ICSRs in VigiBase can automatically be identified with VigiMatch, an algorithm that uses a statistical model to score pairs of reports, taking into account the amount of matching and mismatching information (Tregunno et al., 2014). However, despite this, the final dataset can still contain some duplicates of reports.

It is important to note that the information in VigiBase comes from a variety of sources, and the likelihood that the suspected ADR is drug-related is not the same in all cases (Uppsala Monitoring Centre, 2019).

### Data Analysis

Descriptive analyses in Excel 2013^®^ were used to determine general characteristics of the reports in the broad data set, including total number of reports, reporting country and patient characteristics. As spontaneous ADR for reports for herbals can contain multiple suspect drugs, including conventional medicines, as co-reported suspects, subsequent analyses were undertaken based only on reports with a single suspect herbal drug; these reports were extracted with R Software for Statistical Computing version 3.3.2 (2016-10-31) (The R Foundation, 2019).

From these single suspect herbal reports, reports categorized as “serious” (The Council for International Organizations of Medical Sciences (resulting in death, or is life-threatening; requires inpatient hospitalization or prolongation of existing hospitalization; results in persistent or significant disability or incapacity; results in a congenital anomaly (birth defect); or is otherwise “medically significant”) (CIOMS), 2001) were extracted. The top 10 most commonly reported herbal drugs and most commonly reported ADRs were analyzed. The analysis used the MedDRA Preferred Term level(PT) (The International Conference on Harmonisation of Technical Requirements for Registration of Pharmaceuticals for Human Use (ICH), 2017) and the HM name specified as “Preferred Base Name.” As one report can contain multiple ADRs, some reported ADRs may refer to the same patient.

## Results

**Figure 1:**
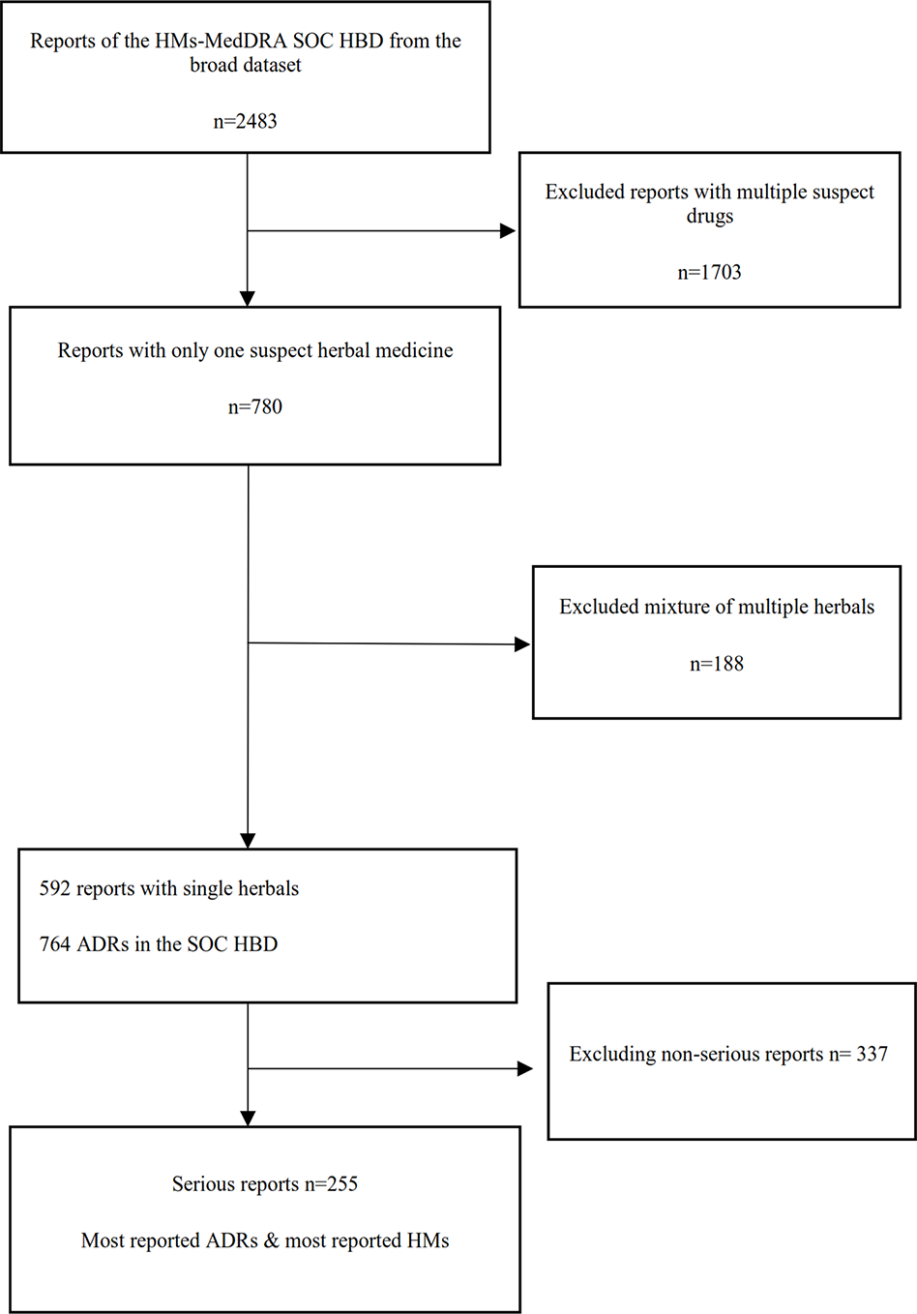
Flowchart of the different analysis steps (HM, herbal medicine; HBD, hepatobiliary disorder; ADR, adverse drug reaction).

A flowchart of the separate analysis steps for HMs that had at least one ADR in the MedDRA^®^ SOC HBD and the resulting sets of data which were analyzed are shown in **Figure 1**.

### Analysis of the Reports of the Broad Dataset

#### General Overview

In total, 2,483 reports for HMs with at least one ADR in the MedDRA^®^ SOC HBD were retrieved from VigiBase, dating from 31-03-1974 to 27-12-2018. This represents 0.013% of all reports in VigiBase. These reports were contributed by 49 countries. The number of reports per country varied; the most frequently reporting countries (top 10) contributed 82.6% (n = 2,051) of the total number of reports, and Japan submitted most reports (n = 569; 22.9% of total), see **Table 1**. The number of reports submitted annually shows an increasing trend, with a peak in 2011 (n = 293) reports.

**Table 1:** Number of reports for herbal drugs submitted by the reporting countries.

Country	Number of reports	Country	Number of reports
Japan	569	Turkey	5
France	295	New Zealand	5
Germany	294	Ireland	5
USA	211	Mexico	4
Spain	134	Viet Nam	3
Australia	130	Uruguay	3
UK	117	Brazil	3
Switzerland	114	Greece	3
India	95	Croatia	2
Singapore	92	Iceland	2
Morocco	70	Ghana	2
Korea, Republic of	63	Kenya	2
Canada	53	South Africa	2
Malaysia	40	Nigeria	2
Norway	36	Nepal	1
Sweden	23	Finland	1
China	20	Uganda	1
Netherlands	14	Saudi Arabia	1
Italy	13	Slovenia	1
Thailand	12	Iraq	1
Poland	8	Hungary	1
Portugal	7	Russian Federation	1
Belgium	7	Argentina	1
Austria	7	Venezuela, Bolivarian Republic of	1
Denmark	6		
**Total 2,483**			

#### Description of the Reported Cases

In total, 2,483 reported cases describing 46,447 ADRs were extracted from VigiBase for all SOCs. Of these, 13,738 ADRs concerned the SOC HBD. Most reports (n = 1032; 41.6%) were initially submitted by physicians; hospitals contributed 12.5%, pharmacists 8.3%, general practitioners 7.7%, other health professionals 7.1%, consumers or other non-health professionals 4.7%, and specialist physicians 4%. For 332 reports (13.3%), the reporter was not known, or more than one profession was mentioned. In the reports, 63% of the patients was female, 35% male, and in 2%, the gender was unknown. The mean reported age for women was 51 years (Median age 52 years) and mean age for men was 50 years (Median age 52 years). The reported age in one report was 220 years; this report was excluded from the analyses.

### Results of the Analysis for Single Suspect Herbal Medicines

#### General Overview

For most of the 2,483 reports, more than one herbal drug was reported as the suspect drug; 780 (31.4%) reports concerned only one suspect HM. However, for 188 reports, the single suspect herbal preparation contains more than one herbal ingredient; these reports described a total of 335 ADRs. From the products containing multiple herbals Iberogast*^®^* (which contains *Angelica archangelica, Carum carvi, Chelidonium majus, Glycyrrhiza glabra, Iberis amara, Matricaria recutita*, *Melissa officinalis, Mentha piperita, Silybum marianum)* was the most frequently reported (n = 10 reports) herbal preparation.

For 592 reports with one suspect HM, the suspect herbal product concerned a single herbal species. Further analysis, reported below, was performed for these reports.

#### Most Commonly Reported Single Suspect Herbals

**Table 2** lists the 10 most commonly reported HMs (by Preferred Base Name) reported in association with HBD SOC ADRs. The largest group, comprising 243 reports describing 309 ADRs, represents HMs or products in the database categorized as unspecified herbal *[coded as Unspecified herbal, Ayurvedic preparation NOS, Herbal extract NOS, Herbal NOS, herbal preparation, unspecified herbal and traditional medicine, unspecified traditional medicine, herbal pollen NOS traditional medicine (Group NOS)].*

**Table 2:** Ten most frequently reported single suspect HMs and number of ADRs per HM.

	Herbal medicine	Number of reports	Number of ADRs
1	Group non specified herbal drugs (nos)	243	309
2	*Chelidonium majus*	38	61
3	*Cimicifuga racemosa*	31	49
4	*Camellia sinensis*	21	25
5	*Piper methysticum*	17	23
6	*Valeriana officinalis*	16	19
7	*Hypericum perforatum*	13	17
8	*Teucrium chamaedrys*	12	13
9 = 10	*Senna siamea*	7	10
9 = 10	*Serenoa repens*	7	9
	Total	**405**	**535**

ADRs, adverse drug reactions; HM, herbal medicines.

#### Most Commonly Reported ADRs

The 592 reports for single suspect herbal preparations described a total of 1,368 ADRs. Since one drug, including herbal drugs, can be associated with multiple ADRs, and as these can relate to different organ systems, not all reported ADRs are within the HBD SOC. From the 1,368 reported ADRs, 764 MedDRA preferred terms were in the SOC HBD. Among these reports, jaundice was the most reported ADR (26%) for reports concerning single suspect herbal drugs. The 10 most commonly reported HBD SOC ADRs are listed in **Table 3**.

**Table 3:** Ten most commonly reported HBD SOC ADRs for reports with single suspect herbal medicines.

	Reported adverse drug reaction (MedDRA PT name)	Number	Percentage (%)top 10/total n = 764
1	Jaundice	146	26.4/19.1
2	Hepatitis	115	20.8/15.1
3	Hepatic function abnormal	90	16.3/11.8
4	Drug-induced liver injury	36	6.5/4.7
5 = 5	Liver injury	29	5.2/3.8
5 = 6	Acute hepatic failure	29	5.2/3.8
5 = 7	Hepatic failure	29	5.2/3.8
8	Hepatitis cholestatic	28	5.0/3.7
9 = 9	Liver disorder	26	4.7/3.4
9 = 10	Hepatitis acute	26	4.7/3.4
	Total	**554**	**100**

ADRs, adverse drug reactions; HBD, Hepatobiliary Disorder; SOC, System Organ Class.

### Results of the Analysis for “Serious” Reports Related to Single Suspect Herbal Medicines

#### General Overview

In total, 255 (43%) of the 592 reports for single suspect HMs were classified as serious. These reports concern 55 different HMs. For 127 reports, the herbal drug is specified (unspecified for 128 reports). In 119 reports (46.7%) the indication was not filled in or PT “drug/product use for unknown indication” was reported. In the reports with a known indication; (unspecified) jaundice was reported 46 times (18%). The top 10 reported indications concern obesity (reported 4 times (1.6%)), menopause (reported 4 times (1.6%)), diabetes and abnormal weight gain (reported 3 times each (1.2%)), migraine, rheumatoid arthritis, exfoliative dermatitis, slimming diet, climacteric discomfort (reported 2 times each (0.8%)). These 255 reports described 540 ADRs, with 329 ADRs coded to the MedDRA^®^ HBD SOC. In men, the most commonly reported ADRs were jaundice (n = 20 reports), drug-induced liver injury (n = 15) and hepatitis (n = 13); among women, these were hepatic function abnormal and hepatitis (n = 20 for each) and jaundice (n = 18). Twenty-two reports were categorized as life threatening (with or without (prolonged) hospitalization) with the reported outcomes; 6 times recovered, one time recovered with sequel, 2 times recovering/resolving, 6 times not recovered, and 7 times not filled in or unknown. One hundred fifty reports were categorized as causing (prolonged) hospitalization where 44 times the patient recovered, 58 times was recovering, 28 times not recovered, 20 times the outcome is not been filled in or was unknown. Sixty-nine times the seriousness were categorized as “other” and 5 times the seriousness criterion was not filled in. In nine reports, the seriousness was categorized as “death.” From those, one report has the outcome not recovered and eight reports described a fatal outcome. For “fatal” reports, the suspect HM was *Rubia peregrina* (n = 2); *Hypericum perforatum*, *Valeriana officinalis*, *Senecio vulgaris*, *Andrographis paniculata*, *Piper methysticum* and *Cephaelis* species were reported as the suspected drug for one report each.

#### Most Frequently Reported Herbals in Reports Classified as Serious

The largest group of HMs among serious reports represented non-specified herbal products. Such products were described in 128 serious reports (50%) for single suspect HM. **Table 4** lists the 10 most commonly reported HMs (after exclusion of non-specified herbal products) with at least more than one report per herbal.

**Table 4:** Ten most commonly reported herbal drugs (after exclusion of non-specified herbal products) among serious reports with ADRs coded to MedDRA HBD SOC.

	Herbal medicine	Number of reports (n >1)	Number of ADRs
1	*Cimicifuga racemosa*	18	26
2	*Valeriana officinalis*	14	17
3	*Camellia sinensis*	9	11
4	*Hypericum perforatum*	8	11
4	*Serenoa repens*	5	7
4	*Pelargonium sidoides*	4	4
7	*Morinda citrifolia*	3	5
7	*Aristolochia fontanesii*	3	3
9	*Glycine max*	2	8
9	*Chelidonium majus*	4	5
9	*Arctostaphylos uva-ursi*	2	4
9	*Hintonia latiflora*	2	4
9	*Viscum album*	2	4
9	*Aloe vera*	2	3
9	*Petasites hybridus*	2	3
9	*Ilex paraguariensis*	2	2
9	*Rubia peregrina*	2	2
9	*Lycium barbarum*	2	2
9	*Silybum marianum*	2	2
	Total	**88**	**123**

ADRs, adverse drug reactions; HBD, Hepatobiliary Disorder; SOC, System Organ Class.

**Table 5** shows the ten most reported serious hepatic ADRs for the HMs (single suspect) listed in **Table 4** (after exclusion of the “non-specified HMs” group).

**Table 5:** Ten most reported serious hepatic ADRs for the HMs (single suspect) listed in **Table 4** (non-specified HMs = Group NOS).

Herbal medicine															
Reported reaction	Group nos	Valeriana officinalis	Cimicifuga racemosa	Camellia sinensis	Glycine max	Serenoa repens	Hypericum perforatum	Chelidonium majus	Hintonia latiflora	Aloe vera	Aristolochia fontanesii	Arctostaphylos uva-ursi	Petasites hybridus	Viscum album	Total
1.Drug-induced liver injury	21	10					1	2		1					35
2.Jaundice	16		3	2		1	2	1	2	1				2	30
3.Acute hepatic failure	21	1											1		23
3.Hepatic function abnormal	22				1										23
4.Hepatitis	13		2	3					1		3				22
5.Liver disorder	18						1								19
6.Liver injury	9		4					1				1			15
7.Hepatitis cholestatic	8	1		2									1		12
8.Hepatitis toxic	4			2		1			1	1		1			10
9.Cholestasis			2		7										9
10.Hepatitis acute	3			2		2						1			8
Total	135	12	11	11	8	4	4	4	4	3	3	3	2	2	206

ADRs, adverse drug reactions; HM, herbal medicine.

## Discussion

This study explored the numbers and characteristics of hepatic ADR reports associated with HMs submitted to the global ICSR database VigiBase.

This analysis extracted 2,485 ICSRs for preparations coded as HMs that had at least one ADR in the MedDRA^®^ HBD SOC. These reports have been contributed by 49 countries since the 1970s; however, they may represent only a small proportion of the cases of liver injury associated with use of HMs. Under-reporting of ADRs associated with HMs is probably an even greater issue than for conventional medicines (Barnes, 2003; Walji et al., 2009; Skalli and Jordan, 2017; ). Often, healthcare professionals and users of HMs are unaware that spontaneous reporting systems accept reports associated with HMs and are unsure how to identify ADRs related to HMs. Also, many patients do not tell healthcare professionals that they use HMs, or may report “product complaints” to individuals, such as healthfood-store staff, outside the reporting framework (Walji et al., 2009). Thus, ADRs may go undetected and unreported to the formal system (Walji et al., 2009).

Under-reporting is a problem in all spontaneous reporting systems, and it can vary between countries: not all countries have been a part of the WHO Programme for International Drug Monitoring for the same amount of time, and some are selective about the type of reports they submit (Farah et al., 2000). Given the wide availability, use and acceptance of TCMs in China (Liu et al., 2015), the under-representation of ADR reports associated with HMs contributed from China in VigiBase demands comment. In China, ADRs are reported to the regional centers and then forwarded to the national center. By the end of year 2013, the national system had collected >6.6 million reports. Numbers of ADR reports and ADR reporting rates for China have shown an increasing trend in recent years (Guo et al., 2015); Reports relevant to TCM increased from 13.3% in 2009 to 17.3% (Guo et al., 2015). However, the proportion of serious reports, as well as the proportions of reports contributed by consumers and pharmaceutical companies, remain low; importantly, in the context of HMs, a greater focus should be placed on reporting for TCMs, particularly TCM injections, some of which have been associated with serious ADRs (Guo et al., 2015). Also, these reports are not all represented in VigiBase. In 2018, approximately 5% of all reports in VigiBase were from China (Ploen, 2018) and our current analysis shows that China is not represented in the top 10 reporting countries for HMs with an ADR in the SOC HBD. From the literature, however, it is known that herbal TCM products can cause hepatotoxicity. An analysis of reported cases found numerous specified herbal TCM products with potential hepatotoxicity and, for several of these, causality seemed likely after further assessment (Teschke, 2014). Clinical outcomes of herbal TCM-induced hepatotoxicity can be serious, including liver transplantations or death (Wang et al., 2018b). The same issue (low numbers of reports for herbal hepatotoxicity in VigiBase) exists for other countries, for example, in Africa and India where the use of HM is also widespread (Skalli and Bencheikh, 2015; Gupta et al., 2017).

To investigate the association between HMs and reported HBD in a (spontaneous) reporting database such as VigiBase, comprehensive causality assessment is necessary (Meyboom et al., 1997). For HM-induced HBD cases, causality assessment should consider: confounding variables related to the documentation of the herbal product and the clinical course of the adverse event; hepatotoxicity and re-exposure criteria; temporal association; concomitant medication and alternative explanations, with special attention to pre-existing diseases of the liver, bile ducts and the pancreas (Teschke et al., 2013). The possibility of adulteration and/or contamination should also be considered (Wang et al., 2018a). Use of tools, such as RUCAM (Roussel Uclaf Causality Assessment Method) and updates, which provides a quantitative assessment of causality for cases of DILI and HILI, may be appropriate (Danan and Teschke, 2015).

For reports with multiple suspect drugs, including those with conventional medicines as co-suspected drugs, determining causality for the reported ADRs is more difficult. For this reason, the in-depth analysis focused on reports with a single herbal preparation as the suspect drug. However, such preparations can still comprise multi-herb mixtures, as described. A limitation of our approach is that many multi-herb preparations described in reports of HBD were excluded from the analysis, yet these products may pose the same, or an even greater, risk of causing HBD as do single-herb preparations. In the dataset, the HMs concerned comprised both finished (manufactured) herbal preparations (some with commercial names) and raw (crude) herbal materials. Further, data on the part of the herb used are not available in this dataset. This information is important as, for some plant species, different parts of the plant are used medicinally; the profile of chemical constituents in different in different plant parts, therefore they are considered to be different herbal drugs. Thus, we cannot exclude the possibility that the hepatotoxicity is related to a “wrong” part of the plant being used. Also, for finished products, we cannot exclude the possibility that counterfeit herbal ingredients were used.

This analysis found that, for a substantial portion of the HM reports in VigiBase, the preparations described are coded as “unspecified herbals”; this presents an important limitation in undertaking causality assessment for individual case reports or case-series and prevents use of these reports for signal detection purposes. Further, it can be challenging to store reports on “unspecified herbals” in a database without the fixed structure that is in place for regular drugs, with registered proprietary names, ingredients and the possibility to code on different levels. Using correct nomenclature is essential in order to precisely identify the herbal substance(s) implicated in ADR reports in a PV database, also for HMs where this can be complex such as TCM (Wu et al., 2007). The use of scientific binomial names, including botanical authority, is essential, in addition to naming the plant part used and preparation method in the reports, and subsequently, in VigiBase (Farah et al., 2000; Farah et al., 2006;Dauncey et al., 2019). However, for several reasons, it is often not possible to assign this information: ADR reports submitted by HMs’ users and health professionals typically use common names for HMs (e.g., “echinacea”), which cannot be coded to a particular plant species and plant part; the herbal preparation used may be an unnamed traditional herbal formula comprising crude (e.g., dried, fragmented) herbal drugs; manufacturers’ product labels also do not precisely define the plant species and parts used for herbal ingredients.

For PV centers that use the WHO Drug Global^®^ dictionary, the Herbal ATC (Anatomical Therapeutic Chemical), or HATC, coding system is available. The HATC classification aims to provide a scientiﬁc framework for a harmonized, global nomenclature and therapeutic classiﬁcation of herbal substances and combinations of them (the Uppsala Monitoring Centre, 2017). However, the system has major limitations, and is not universally used. Limitations include that HMs may contain multiple ingredients and it is not always possible to identify them all. In addition, the HATC classification was developed for the whole plant, but not for a given part of the plant, which may be coded in several places in the HATC classification. Also, HMs from reporting countries may have only the vernacular name in the label of the product. Since VigiBase contains reports that have been transferred from local PV databases, it is essential that in the reporting, coding, assessing, storing, and transferring of HM reports essential information on the product is recorded.

Single suspect HMs for which serious hepatic reactions were most frequently reported were *Cimicifuga racemosa (Actea racemosa,* black cohosh*)*, *Valeriana officinalis, Camellia sinensis*, and *Hypericum perforatum*. Black cohosh (*Actea racemosa*) has previously been associated with hepatotoxicity (Whiting et al., 2002; van de Meerendonk et al., 2009; Teschke, 2010;Teschke et al., 2011). However, cases described in the literature have confounding variables as well as poor quality data on the product implicated, ADR description, temporal association, co-morbidities, among others (Teschke, 2010) making causality assessment challenging (Teschke et al., 2009). Case descriptions of hepatotoxic reactions associated with *Valeriana officinalis* are more scarce (Cohen and Del, 2008; Vassiliadis et al., 2009;Douros et al., 2016). Also, for *Hypericum perforatum,* few cases of hepatotoxicity have been described (Dominguez Jimenez et al., 2007;Agollo et al., 2014). In a publication by Mazzanti et al, *Camellia sinensis* has been associated with nineteen cases of hepatotoxicity related to the consumption of herbal products containing green tea (Mazzanti et al., 2015). The same plant, *Camellia sinensis*, is used to produce all types of tea, and the differences among the various types arise from the different processing steps that are used. Based on the degree of fermentation, tea can be classified as black, green, white, or oolong tea. Of these, black tea is the most or fully fermented tea (Jolvis Pou, 2016).

In addition, the increasing international trade in HMs raises concerns around the safety and efficacy of these products (Walker and Applequist, 2012). There might be adulterated material in the supply chain of raw/crude herbal ingredients, or falsification in herbal products (Mishra et al., 2016). In this case, liver injury in relation to counterfeit herbal products may be due to the presence of plant species not declared on the product label. Strict legislation and quality control are needed. In addition, the development of crude drug repositories to maintain authentic botanicals as reference standards is essential (Srirama et al., 2017). The Royal Botanical Garden Kew for instance has a Chinese Herbal Medicine Authentication centre (Royal Botanical Gardens Kew, 2019).

### Strengths and Limitations

A strength of this study is the advantage of presenting information from the global WHO ICSR database, where reports from all countries belonging to the international PV program are concerned. However, in VigiBase, the likelihood that the reported event was caused by the medicine varies from report to report. Some countries collect only suspected ADRs with at least a possibility of a causal relationship between the drug and reported event, while for example the United States, contributing almost half of the reports in VigiBase, collects *“…any adverse event associated with the use of a drug in humans, whether or not considered drug related…”* (GPO US Government Publishing Office, 2019). Because the data extracted from VigiBase itself had limitations in documentation, as mentioned above in our discussion, and the narrative of the individual ICSRs was not available, no causality assessment for individual cases was performed. The MedDRA codes for HBD do not provide (all) diagnostic information that might be present in the report. In addition, information on the dosage used in the reports was very incomplete and from our analysis of the data also seems incorrect on instance. Therefore, we decided not to use the data as it, unfortunately, would not be of value to the article. Considering the reported indications, jaundice was reported as the indication in 18% of the serious reports. However, it is difficult to ascertain if coding of the indications was done correctly at local PV centers.

As general limitations, we also point to the problem of under-reporting ADRs in relation to HMs (Barnes, 2003; Walji et al., 2009; Skalli and Jordan, 2017), and the fact that methods and tools for causality assessment and signal detection have mostly been developed for conventional medicines and can be applied with difficulty to HMs. These need to be better adapted for a better and efficient phyto/herbovigilance.

## Conclusion

This study explored reports of HBD associated with the use of HMs in the global ADR database VigiBase. Almost 2,500 reports for HMs and with at least one ADR coded to the MedDRA^®^ SOC HBD were retrieved from VigiBase; this number represents 0.013% of all reports in VigiBase. Many countries were under-represented as contributors of reports, particularly those where use of herbal and other traditional medicines is a widely accepted or, in some cases, the only, form of healthcare available to millions of people. Substantial effort is required at the local, national, and international levels to raise awareness among users of HMs, herbal and traditional medicine practitioners, and healthcare professionals of the importance of considering, identifying and reporting cases of suspected ADRs associated with use of herbal and traditional medicines, and to facilitate access to reporting mechanisms, particularly in countries where a majority of the population relies on these preparations for their health and well-being.

Of the HBD SOC HM reports, around 25% concerned a single herbal species as the suspect “drug.” Substantial issues with coding of the suspect drugs were found. For single suspect HMs with multiple reports of HBD classed as “serious,” in-depth causality assessment of the cases is needed to draw conclusions on the strength of the relationships.

## Data Availability Statement

The datasets for this manuscript are not publicly available because of the UMC data protection policy. The first author is responsible for the data being handled according to the UMC guidance for use of VigiBase data in studies. Requests to access the datasets should be directed to FH, f.vanhunsel@lareb.nl.

## Author Contributions

FH, SK, SS, AK, J-BW, and JB contributed conception and design of the study. FH organized the dataset. SK, FH, and LT performed the (statistical) analysis; FH and SK wrote the first draft of the manuscript with input of all other authors. All authors contributed to manuscript revision, read, and approved the submitted version.

## Conflict of Interest

The authors declare that the research was conducted in the absence of any commercial or financial relationships that could be construed as a potential conflict of interest.
